# Vehicles for Drug Administration to Children: Results and Learnings from an In-Depth Screening of FDA-Recommended Liquids and Soft Foods for Product Quality Assessment

**DOI:** 10.1007/s11095-022-03208-y

**Published:** 2022-03-01

**Authors:** Lisa Freerks, Wenke Sucher, Marie-Josefin Tarnow, Carolin Eckert, Sandra Klein

**Affiliations:** Department of Pharmacy, University of Greifswald, Institute of Biopharmaceutics and Pharmaceutical Technology, Center of Drug Absorption and Transport, Felix Hausdorff Straße 3, 17489 Greifswald, Germany

**Keywords:** Bioavailability, Compatibility, Drug stability, Food effect, Pediatric drug product

## Abstract

**Purpose:**

Mixing with liquids or soft foods is a common procedure to improve acceptability of oral medicines in children but may affect drug stability and the *in vivo* performance of the administered drug product. The aim of the present study was to obtain an overview of the variability of critical attributes of commonly used vehicles and to identify which vehicle characteristics need to be considered when developing *in vitro* methods for evaluating product quality.

**Methods:**

One product of each vehicle listed in the FDA draft guidance “Use of Liquids and/or Soft Foods as Vehicles for Drug Administration” was analyzed with regard to composition, calorific content and physicochemical properties.

**Results:**

The studied vehicles show wide variability, both in composition and physicochemical properties. No correlation was observed between vehicle composition and physicochemical properties. Comparison of results of the present study with previously published data also provided variability in physicochemical properties within individual vehicle types.

**Conclusions:**

To identify acceptable (qualified) vehicles for global drug product labeling, it is important that the vehicles selected for *in vitro* compatibility screening reflect the variability in composition and essential physicochemical properties of the vehicles recommended on the product label, rather than relying on results obtained with a single vehicle of each type. Future activities will focus on the development of standardized dosing vehicles that can represent key vehicle characteristics in all their variability to ensure reliable risk assessment.

## Introduction

Despite the growing awareness of the need for age-appropriate formulations, the number of authorized pediatric medicines available is still far behind that of adult medicines. Since there is still a huge need for child-appropriate dosage forms, manipulation of dosage forms is a common practice prior to drug administration to children. Manipulation is defined as the process of physically altering dosage forms in order to provide the prescribed dose and to enhance patient acceptability [1, 2]. Manipulation of dosage forms can be carried out in different ways depending on the type of the dosage form and the preferences of the caregivers and the pediatric patients, respectively. In the case of solid oral dosage forms, common manipulation practices are for example opening of capsules and splitting or crushing of tablets [1]. Moreover, the manipulated dosage forms as well as other solid oral dosage forms, such as granules or mini tablets, are often mixed with small amounts of soft foods or liquids, in this context referred to as vehicles, prior to drug administration to children with the aim of improving palatability and swallowability [2, 3].

So far, there are no precise rules for the joint administration of oral drug products and vehicles. Recommendations regarding suitable vehicles, that become part of the summary of product characteristics (SmPC) and patient information leaflet (PIL) of an individual drug product, are usually made on a product-specific basis. While the suitability of the vehicles listed in SmPCs and PILs has been proven in compatibility studies as part of their authorization and co-administration with them can therefore be considered safe, the daily practice of dose administration can be quite different because, as for instance stated by Martir et al*.,* there is no guarantee that parents, caregiver, and healthcare professionals will adhere to the prescribed procedure [4]. Depending on the type of soft food or liquid, dosing vehicles can differ significantly in calorie content, fat:protein:carbohydrate ratios and physicochemical properties. Moreover, even vehicles of the same type can differ due to manufacturing variations related to seasonal, regional, and climatic conditions, or simply by manufacturer and brand [5–8]. In addition, the availability of vehicles, as well as the types of vehicles commonly used can vary significantly around the world [9]. Variations in the type but also the volume of vehicle used to administer the drug product may, however, alter quality and *in vivo* performance of the drug product. Generally, drugs should be mixed with small volumes (5–15 mL) of foods or liquids and then administered immediately. A precise upper limit of the maximum permissible vehicle volume has not been specified [10], but it would be essential to prevent unintended *in vivo* performance of the co-administered drug product.

While the vehicle volumes to be used for administration seem very small at first glance and it would hardly pose a major problem for older children, such as school children and adolescents, whether one administers a dosage form with 5 or 15 mL of a dosing vehicle, it should be noted that this mode of administration is usually relevant for very young children, i.e., infants and preschool children. If one now assumes a very young infant, for example, administration is made to a patient in which the dimensions of the gastrointestinal tract are still very different from those of a school child or adolescent. Since especially in young infants both gastric capacity and the amount of fasted resting gastric fluid are much smaller than in older children [11], one can imagine that even a small amount of vehicle may be sufficient to alter the gastric environment and to some extent temporarily might also affect intraluminal conditions in the small intestine, increasing the likelihood of changes in the *in vivo* performance of the drug product. To avoid such undesired effects, only those liquids and/or soft foods that have been shown not to alter the performance of the drug product and which are considered well tolerated and suitable for use in the targeted patient populations should be considered as vehicles for that specific drug product [10]. For some drug products, compatibility with selected vehicles has already been demonstrated [12–20]. On the contrary, there are also some publications which show that vehicles can have a tremendous impact on the drug product performance [6, 7, 21–23]. Given the enormous variety of liquids and soft foods that could potentially be used as dosing vehicles, the question arises as to what potential impact the selected vehicle may have on the stability of the administered drug as well as the *in vivo* performance of the administered drug product. There is also the question of how best to perform a risk assessment, how many different kinds of vehicles to assess and whether it will be necessary to include different vehicles of the same type in such a study. With the aim of standardizing not only the methodology supporting selection and qualification of the vehicle to be mixed with the drug (product), but also the preparation and use instructions for the drug product vehicle mixture, in 2018 the FDA published the draft guidance “Use of Liquids and/or Soft Foods as Vehicles for Drug Administration: General Considerations for Selection and *In Vitro* Methods for Product Quality Assessments” [10]. According to this draft guidance only those liquids and/or soft foods demonstrated to have no appreciable effect on drug product performance should be proposed as vehicles and the potential impact of a vehicle on drug product performance should be determined by assessing drug product quality attributes, including potency (assay), *in vitro* dissolution/release, and other pertinent attributes when the drug product is used with the proposed vehicle(s) [10]. Regarding the selection of vehicles for compatibility assessments, the draft guidance pays particular attention to the pH value of a potential dosing vehicle. It is stated that “the pH value of proposed liquids and soft foods should be considered before further testing for their compatibility with the intact or manipulated drug product” [10] and a table of commonly used soft foods and liquids including their approximate pH range is presented in Appendix A of the draft guidance.

The pH value of soft foods and fluids is certainly an important parameter, since it can have a major impact on solubility and dissolution rate of ionizable drugs [24]. It can further affect drug stability as well as stability and *in vivo* performance of the drug product. Particularly for enteric coated drug products, vehicle pH and mixing time can be very critical, since exposing such formulations to vehicles with higher pH values could present with the loss of coating integrity which could be accompanied by significant degradation of the drug to be administered [23]. However, it is important to consider whether one should focus on the pH value in isolation when selecting vehicles for initial compatibility studies, or whether one might want to take a closer look at the composition and other properties that possibly could influence the *in vivo* performance of the drug product. The reason for such considerations quickly becomes apparent when taking a closer look at Appendix A of the FDA draft guidance, where quite detailed pH ranges for commonly used liquids and soft foods are listed. If, for example, one now wanted to select a vehicle with a pH value within a pH range of 4.4–5.2, the choice would be between buttermilk, yogurt, mashed bananas and maple syrup, i.e., four vehicles for which even without the availability of further detailed information differences in both composition and physicochemical properties can be anticipated. *In vitro* compatibility studies as well as *in vivo* studies with these vehicles might thus well provide different results despite the same pH value. Therefore, when it comes to ensuring that the quality of the drug (product) is maintained when mixed with a vehicle and administered to the target patient population, it is advisable to pay attention also to other characteristics besides the pH of the vehicle from the very beginning. If possible, the detailed composition of the vehicle in terms of fat:protein:carbohydrate ratio, as well as other physicochemical properties, should be considered in order to allow a good pre-selection of vehicles for the assessment of a specific drug product. The goal should be an appropriate risk assessment procedure ensuring the determination of acceptable (qualified) vehicles for drug labeling, but, especially in pediatric drug development, without taking so much time that marketing of the product is unnecessarily delayed.

With the aim of obtaining an initial overview of the variability of the critical attributes of commonly used dosing vehicles and of enabling an estimation of which vehicle properties should be considered when qualifying vehicles for drug labeling, the objective of the present work was to examine one product each of the liquid and soft foods listed in the FDA draft guidance with regard to composition and physicochemical properties.

## Materials and Methods

### Materials

The products investigated in the study are listed in Table 1. Except for unstrained baby food, one product of each vehicle listed in the FDA draft guidance was examined. The products were purchased from local supermarkets or online shops in various countries such as Germany, the United States (USA), the United Kingdom (UK) and Thailand. All chemicals used for physicochemical characterization were of analytical grade and purchased commercially.

**Table 1:** Products investigated in the study

Soft food / liquid	Brand mark	Manufacturer
Apples (puree)	Handmade apple puree from 300 g peeled Pink Lady apples and 200 g demineralized water	Pink Lady, Lidl Stiftung & Co.KG, Neckarsulm, Germany
Apple juice	Alosa, Apfelsaft klar	Brands & Systems BSG GmbH, Hamburg, Germany
Applesauce	Babylove, Apfel pur	dm-drogerie markt GmbH + Co. KG, Karlsruhe, Germany
Bananas (puree)	Ella’s kitchen, bananas	Ella’s kitchen, Henley-on-Thames, UK
Buttermilk	Müller, Reine Buttermilch	Molkerei Alois Müller GmbH & Co KG, Aretsried, Germany
Carrots (puree)	dm Bio, Karotte Pur	dm-drogerie markt GmbH + Co. KG, Karlsruhe, Germany
Chocolate pudding	Dr. Oetker, Sahne Pudding Vollmilch Schokolade	Dr. August Oetker, Bielefeld, Germany
Coconut milk	AROY-D, Coconut Milk	Thai agri Foods Public Company limited, Samutprakarn, Thailand
Cranberry juice	Alnavit, Bio Cranberry Muttersaft	Alnavit GmbH, Bickenbach, Germany
Drinking water	Humana, Baby-Wasser	Humana Vertriebs GmbH, Bremen, Germany
Fruit jelly	Smuckers, Concord Grape Jelly	The J.M. Smucker CO., Orrville, USA
Fruit jam	Kroger, Squeezable Jelly Strawberry	Strawberry jam, The Kroger Co., Cincinnati, USA
Grapefruit juice	albi, Pink Grapefruit	albi GmbH & Co. KG, Bühlenhausen, Germany
Honey	Langnese, Flotte Biene, Sabienchens Honig	Langnese Honig GmbH & Co. KG, Bargteheide, Germany
Infant formula	Nestlé, Beba Pro 1 Anfangsmilch	Nestlé Nutrition GmbH, Frankfurt, Germany
Maple syrup	Tomahawk, Original Kanadischer Ahornsirup	Dockhorn & Co. Import–Export GmbH, Hamburg, Germany
Milk	Weihenstephan, Haltbare Milch 3.5% Fett	Molkerei Weihenstephan GmbH & Co. KG, Freising, Germany
Orange juice	Hohes C, Orange	Eckes-Granini Deutschland GmbH, Nieder-Olm, Germany
Orange marmalade	Valensina, Orange, Fein passiert	Zentis GmbH & Co KG, Aachen, Germany
Peanut butter	Nick, Peanut-butter creamy	Rila Feinkost-Importe GmbH & Co. KG, Stemwede-Levern, Germany
Pineapple juice	Edeka, Ananas Direkt Saft!	Edeka Zentrale Stiftung & Co. KG, Hamburg, Germany
Rice pudding	Ambrosia, Rice Pudding	The Premier Foods Group, London, UK
Soybean milk	dm Bio, Soja Drink Natur	dm-drogerie markt GmbH + Co. KG, Karlsruhe, Germany
Strawberries	Rewe Beste Wahl Erdbeeren, Ganze Früchte tiefgefroren	Rewe "Beste Wahl", Köln, Germany
Yogurt	Nestlé, LC1 Pur, Joghurt mild 3.5% Fett	Lactalis Nestlé Frischprodukte Deutschland GmbH, Kehl, Germany

### Preparation of the vehicles prior to physicochemical characterization

Most of the vehicles listed in the FDA draft guidance are commercially available liquid or semisolid foods characterized by a more or less homogeneous state with varying consistencies. Except for apple puree and strawberries, commercially available soft foods and liquids were studied. Apple puree was prepared from fresh apples. For this purpose, 300 g of peeled and sliced apples and 200 g of demineralized water were placed in a 1-L beaker and heated on a hot plate with occasional stirring (IKA^®^ RCT basic, IKA-Werke GmbH & Co. KG, Staufen, Germany), first to 60 °C for 15 min and then to 80 °C for another 10 min. The mixture was subsequently homogenized with a stick blender (P8-RM-SB, Dirk Rossmann GmbH, Burgwedel, Germany) for 30 s. To investigate the physicochemical properties of strawberries, frozen strawberries were thawed to room temperature and homogenized with a stick blender for 30 s.

### Composition of the vehicles investigated in the study

Specific product information, such as the fat:protein:carbohydrate ratio and calorific content of the individual vehicles, was taken from the product labels and documented for further evaluation. Since the apples used to prepare the apple puree were not labeled, the detailed product information was taken from the website of the producer [25].

### Physicochemical characterization of the vehicles

The physicochemical properties, i.e., the density, pH, buffer capacity, osmolality, surface tension and the viscosity/rheological properties of all vehicles listed in Table 1 were measured as described in [5] if not stated otherwise. With the exception of rheological measurements (n = 3), all tests were performed in sextuplicate (n = 6), and the results were expressed as mean ± standard deviation (S.D.). Except for osmolality, parameters were recorded at 25.0 °C ± 0.5 °C.

#### Density

The density of fluids was measured using a digital density meter (Type DMA 10, serial no: 1030, Anton Paar GmbH, Graz, Austria). The density of semisolid foods was determined using a 10 mL measuring cylinder by weighing the measuring cylinder on an analytical balance (MC1 Laboratory LC 620 P, Sartorius AG, Göttingen, Germany) before and after adding 10 mL of vehicle. The densities of the semisolid foods were used to calculate the accurate vehicle masses required for the experiments described in the following sections.

#### pH value and buffer capacity

The pH value was measured using a calibrated pH-meter (HI 99,161, with electrode FC202D, HANNA Instruments Deutschland GmbH, Vöhringen, Germany). In order to determine the buffer capacity of fluids, defined volumes were tempered in a laboratory shaker (IKA^®^ KS 3000i control, IKA-Werke GmbH & Co. KG, Staufen, Germany) and quantified by potentiometric titration using different concentrations of hydrochloric acid (0.01 M, 0.02 M, 0.03 M, 0.05 M, 0.1 M or 0.2 M HCl, Merck KGaA, Darmstadt, Germany). For measuring the buffer capacity of the soft foods, a defined mass was mixed with demineralized water and then titrated as described for the fluids. For calculating the buffer capacity according to USP, the volumes of hydrochloric acid were determined which resulted in a change of the pH value by one unit.

#### Osmolality

The osmolality was measured via freezing point depression method (semi-micro osmometer K-7400, Knauer Wissenschaftliche Geräte GmbH, Berlin, Germany). Where possible, osmolality of the vehicles was measured directly without any further preparatory treatment. Since the osmolality of semisolid foods and some of the fluids could not be assessed directly, a set of four dilutions was prepared with demineralized water. These dilutions were mixed for 1 min using a Vortex mixer (VWR Reagenzglasschuettler, VWR International GmbH, Darmstadt, Germany) and then centrifuged for 15 min at 4,000 rpm (Eppendorf Centrifuge 5702 R, Eppendorf AG, Hamburg, Germany). 150 µL of the aqueous phase were then added to a 1.5 mL SafeSeal reaction tube (Sarstedt AG & Co. KG, Nürnbrecht, Germany) and each dilution was measured six times. Since a positive correlation between the dilution and the measured osmolality was observed (regression coefficient (R^2^) > 0.97 for maple syrup and R^2^ ≥ 0.99 for all other vehicles requiring dilution), the osmolality of the undiluted vehicle was extrapolated via linear regression.

#### Surface tension

The surface tension of the vehicles was determined with a ring tensiometer (K11, Krüss GmbH, Hamburg, Germany). For determining the surface tension of semisolid foods, a set of four different aqueous dilutions was prepared. Each dilution was measured in sextuplicate. Since there was no big change in the surface activity of the different dilutions, it was assumed that surfactant concentrations were above the critical micelle concentration (CMC) and the surface tension of the aqueous phase of the vehicles was calculated as the mean ± standard deviation of all measured values (n = 24).

#### Viscosity

The viscosity of all Newtonian fluids was determined with a suitable Ubbelohde viscosimeter (either type 0c, K = 0.003188 mm^2^/s^2^; type I, K = 0.009623 mm^2^/s^2^ and K = 0.010080 mm^2^/s^2^; or type II, K = 0.099370 mm^2^/s^2^; calibrated according to DIN 51,562; SI Analytics, Mainz, Germany).

Due to the fruit pulp present in pineapple juice and the high viscosity and non-Newtonian flow behavior of the semi-solid foods, the rheological properties of these vehicles were determined using a cup and bob rotational viscometer (Brookfield DV3T with DIN 87 spindle and ULA-DIN-6Y sample container, Brookfield Engineering Laboratories, Middleborough, USA) applying various shear rates.

## Results and Discussion

With the goal of gaining better insight into the variability of critical attributes of commonly used dosing vehicles, one representative of each of the vehicles listed in the FDA draft guidance was thoroughly investigated. Although grapefruit juice is not recommended as a vehicle at all and honey is not recommended for children under the age of 12, they were included in the study, since dosing vehicles are not just used in pediatric patients, but also other patient populations who are unable to swallow solid oral dosage forms. Results on composition and physicochemical properties of the vehicles listed in Appendix A, which are presented and discussed in the following section will therefore be of interest for non-pediatric applications as well.

### Composition of vehicles investigated in the study

The vehicle composition is an important factor to consider when co-administering a drug product with food or fluids as it can influence stability, solubility, and dissolution rate of drugs. The extent to which the dosing vehicle affects one or more of these parameters depends on many factors, including the drug substance and its formulation, the composition and physicochemical properties of the vehicle, the contact time with the vehicle prior to ingestion, the amount of dosing vehicle used to administer the dosage form and, the physiological conditions in the patient's gastrointestinal tract. The latter in turn depend very much on the patient's age, size, body weight, and possibly also on the patient's disease status. When co-administered with vehicles with a high fat content, poorly water-soluble lipophilic drugs may (partly) dissolve in components of the dosing vehicle, whereas in pure water, they often may not dissolve at all [24, 26]. This could be associated with altered bioavailability, especially in very young children in which the resting volumes of gastric and small intestinal fluid are still relatively small and thus the volume and composition of the co-administered vehicle may have, at least temporarily, a much stronger impact on intraluminal conditions than in older children [11].

However, vehicles can influence the solubilization of drugs not only directly but also indirectly. For example, during digestion the distribution of drugs between the lipid phase and the aqueous phase of the gastrointestinal contents may be altered, which might also influence drug absorption. Such effects would also be expected primarily for lipophilic drugs [27, 28]. In addition to altering intraluminal contents, the co-administered vehicle may have further effects on physiological parameters, such as for instance gastric emptying rate. After food ingestion one of the factors determining gastric emptying rate is the caloric content of the food ingested. Increasing caloric content of the co-administered food or liquid may lead to an increase in the residence time of drugs in the stomach, possibly resulting in a delay in drug absorption [26]. As mentioned before, fasted administration of small amounts (e.g., 5 mL) of dosing vehicles in older children will most likely not result in pronounced vehicle-related food effects, whereas, when co-administering somewhat larger vehicle volumes in very young children, the possibility of such effects should certainly be kept in mind.

**Fig. 1:**
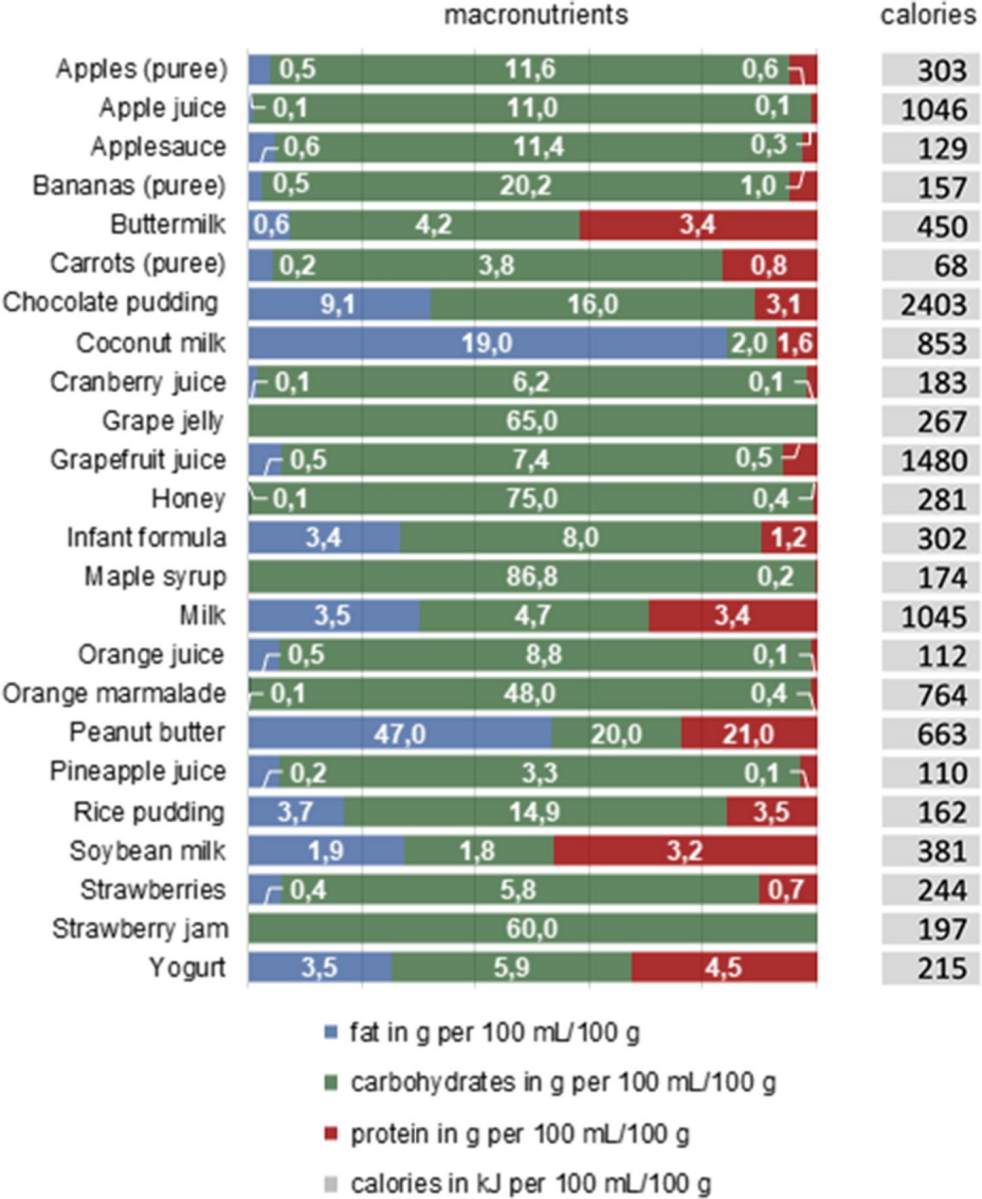
Fat:carbohydrate:protein ratio and calorific content of the vehicles investigated in the study

Figure 1 represents the fat-, carbohydrate- and protein- content as well as the calorific content per 100 mL of fluid or per 100 g of semisolid food, respectively. Most of the vehicles studied are carbohydrate-based, but overall, there is wide variability in both the percentages of fat, carbohydrates, and proteins as well as in the absolute nutritional values of the vehicles. Coconut milk (19% fat) and peanut butter (47% fat), for example, are vehicles with significantly higher fat contents, which should be considered when co-administering these vehicles with lipophilic drugs.

### Physicochemical properties of vehicles investigated in the study

**Fig. 2:**
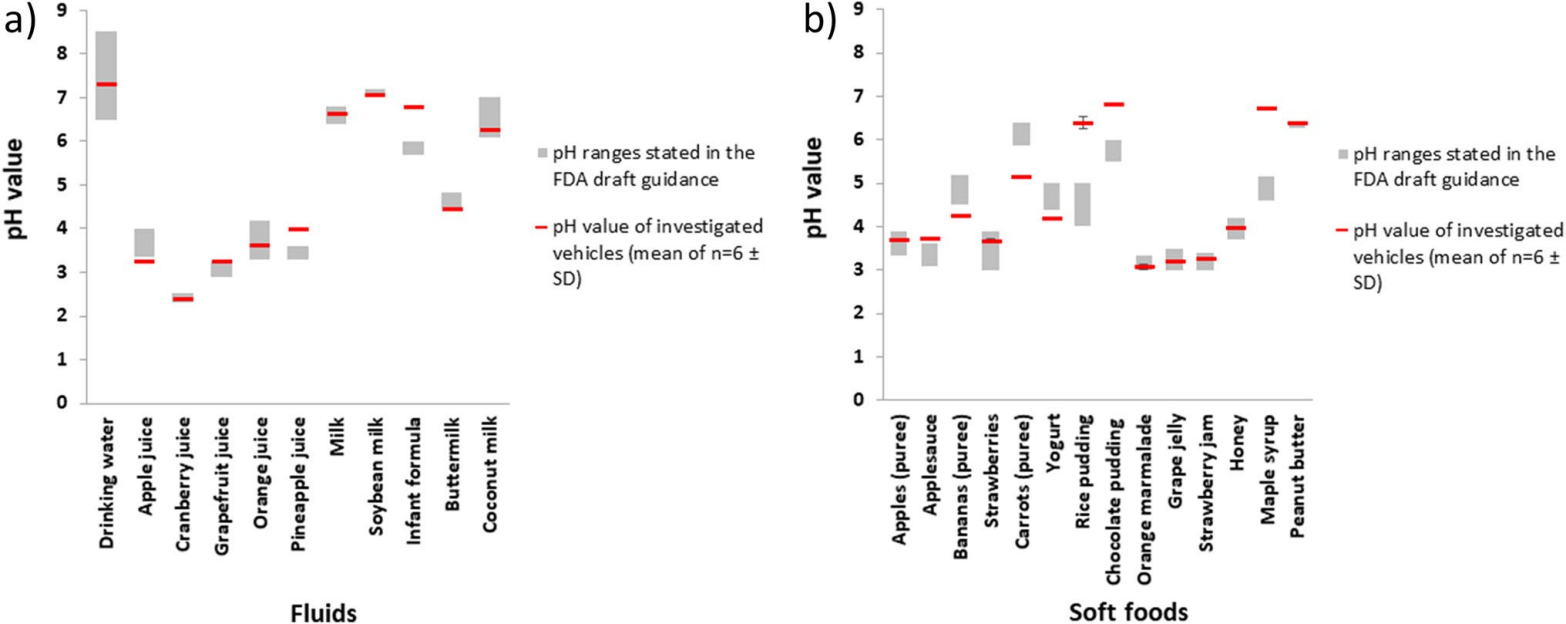
pH values of the vehicles investigated in the study (mean of n = 6 ± S.D.) in comparison to the pH ranges specified in Appendix A of the FDA draft guidance [10]

**Fig. 3:**
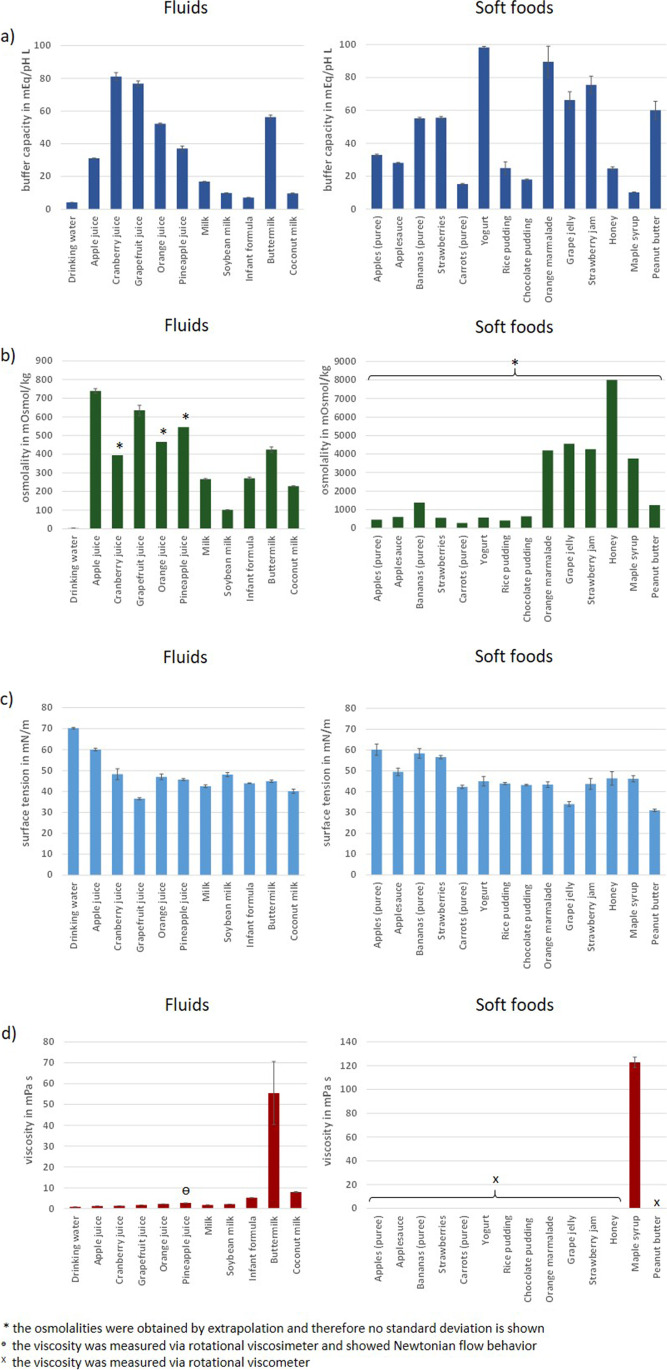
Buffer capacity (**a**), osmolality (**b**), surface tension (**c**) and viscosity (**d**) of the vehicles investigated in the study (mean of n = 6 ± S.D.)

**Fig. 4:**
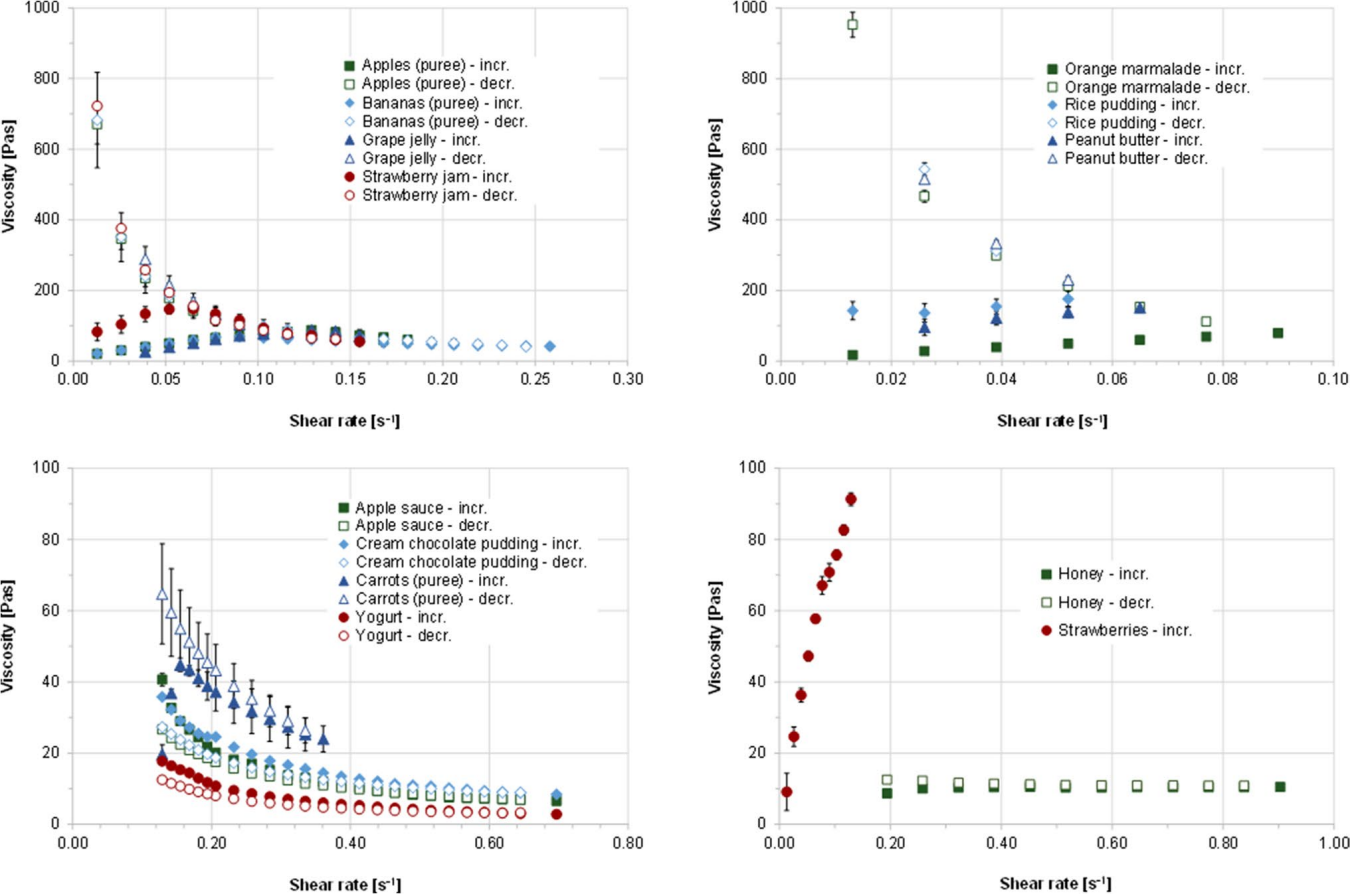
Viscosity profiles of investigated soft foods at 25 °C and increasing and decreasing shear rates (mean of n = 3 ± S.D.)

Each of the physicochemical properties assessed in this study may have an impact on drug stability and drug product performance. The influence of the pH value on the solubility and dissolution rate of ionizable drugs as well as the integrity of enteric coatings has already been mentioned. The pH conditions in the gastrointestinal lumen are subject to a wide variety of factors. If a drug is co-administered with a dosing vehicle in the fasted state, pH and buffering capacity of the co-administered vehicle may have a significant impact on the intraluminal pH conditions, especially in very young children. The surface tension of the vehicle can affect drug release/dissolution, as it affects wetting and thus the effective surface area of the drug available for dissolution and, depending on the CMC of the surfactant present in the vehicle, there might even be a chance for increased drug solubilization immediately after administration. Therefore, vehicles with a low surface tension may contribute to the dissolution and, in individual cases, possibly also the bioavailability of poorly soluble drugs. Although this might be of only minor impact in most cases, vehicle osmolality, by contrast, may impact drug release of dosage forms that release the drug depending on osmotic pressure differences [24]. A high vehicle viscosity as well as an increase in viscosity of luminal contents has proven to negatively affect the dissolution of various drugs as it results in a decreased diffusivity [7, 24]. Moreover, vehicles that contribute to a higher osmolality and/or viscosity of the gastric contents could also affect gastric emptying which is, however, controlled by several additional factors including the caloric content and the detailed composition of gastric contents [29–32]. Based on these considerations, it is obvious that the potential impact of vehicles on rate and extent of oral drug absorption goes far beyond the impact of the vehicle pH, which is why essential differences of typical vehicles were studied in more detail. The physicochemical properties of the vehicles investigated in this study are summarized in Figs. 2, 3 and 4.

Appendix A of the FDA draft guidance provides approximate pH ranges of the different soft foods and liquids regarded as commonly used vehicles. Since many of the listed values are given very precisely, it looks at first glance as if this information is very reliable. At second glance, however, one notices that often only one value is specified with standard deviation or minimum and maximum values being missing, which seems very unlikely given the large variety of food products available. If then following the link provided, which leads to the source of the majority of these data (https://hgic.clemson.edu/factsheet/canning-foods-the-ph-factor/), it turns out that this is a fact sheet specifying how food should be processed for preservation depending on its pH. Since this is anything but a scientific publication, the true origin of the data remains unknown. To determine whether the pH ranges given in Appendix A are representative, the pH of one product/preparation of each of the listed vehicles was measured and compared to the pH (range) reported in Appendix A (Fig. 2). Results shown in Fig. 2 confirmed that most of the vehicle pH values are in the acidic range but indicate that the experimentally determined pH values are partly within the indicated ranges, whereas for some vehicles, such as for instance infant formula, maple syrup, rice pudding etc. the pH can be far outside that range, indicating a high variability of pH values even within the same vehicle type. Already after determining the pH of a single vehicle of each type, it is therefore clear that the pH data from Appendix A cannot be regarded as scientifically sound, which is probably also the reason why the draft guidance states that these are approximate pH ranges. It is likely that also the pH ranges provided here were measured for just one or very few representatives of each vehicle type. The results shown in Fig. 2 clearly indicate that the pH of the vehicle may be subject to considerable variability for various reasons. While the pH of unprocessed foods and liquids can vary depending on variety, origin, and season, it is quite common to adjust the pH of processed foods to optimize the chemical stability of the vehicle as well as other key vehicle properties such as taste, viscosity, and texture. Regardless of the reasons for which the pH may vary, this variability must be taken into account when specifying acceptable (qualified) dosage vehicles in drug product labeling. Of some vehicles, many different subtypes and brands are available, which differ considerably in composition and possibly also in physicochemical properties. Nevertheless, they are often grouped under a collective name. This applies in particular to milk, infant formula, and unstrained baby food, but also to many other liquids and soft foods. In the case of unstrained baby food, no specific food product was tested in the present study, because this general name could not be clearly assigned to a specific kind of vehicle. It is therefore even more interesting that in Appendix A a very narrow pH range of pH 5.95 to 6.05 is specified for unstrained baby food. With regard to the stated pH value, two other vehicles (peanut butter and soy milk) were initially conspicuous, as there is no pH range stated for either of them, but fixed pH values of pH 6.28 for peanut butter and pH 7 for soy milk, respectively. The pH measurement in the present study did indeed provide similar values with a measured pH value of 6.41 for peanut butter and pH 7.06 for soy milk for the products assessed. Whether this is a random incident or whether the pH value of these vehicles is generally subject to only small fluctuations would need to be clarified in further investigations. Overall, results of the pH measurements raise the question of how many vehicles of the same type should be studied to determine adequate and comprehensive ranges of pH values which would be essential for proper risk assessment in the process of identifying acceptable (qualified) vehicles for labeling.

In addition to pH, several other characteristics of the vehicle can affect drug stability and drug product performance. For this reason, buffer capacity, osmolality, surface tension, and viscosity/rheological properties were also investigated. Density is not discussed in detail here, as it was mainly used to calculate food volumes based on the masses of the soft foods. An overview of the properties of all vehicles, grouped into fluids and soft foods, is shown in Fig. 3. Since most of the soft foods either showed non-Newtonian flow behavior or their viscosity was too high for using an Ubbelohde viscosimeter, their flow behavior was measured with a rotational viscometer and is depicted in Fig. 4.

For all physicochemical properties investigated, large differences could be observed among the vehicle types. Overall, surface tension was the parameter with the smallest fluctuations, while strong variability was observed for the other parameters. Expectedly, buffer capacity was lowest for drinking water (0.11 ± 0.00 mEq/pH/L). The highest buffer capacity was measured for yogurt (98.27 ± 0.65 mEq/pH/L), which contains a relatively balanced mix of fat, carbohydrates and proteins, followed by orange marmalade (89.50 ± 9.43 mEq/pH/L), cranberry juice (81.13 ± 2.42 mEq/pH/L), grapefruit juice (76.75 ± 1.64 mEq/pH/L), strawberry jam (75.50 ± 5.24 mEq/pH/L) and grape jelly (66.25 ± 5.07 mEq/pH/L), all of which are very high in carbohydrates, particularly mono- and disaccharides. If one also considers the buffer capacities of the other soft foods and liquids, it becomes apparent that there is no real correlation between the content and ratio of macronutrients and the buffer capacity. Here, it would be necessary to look at the composition of each vehicle down to the smallest detail. As for the buffer capacity, osmolality was lowest for drinking water (4 ± 1 mOsmol/kg), since a drinking water obtained by reverse osmosis was assessed in the study. Soft foods rich in short-chain carbohydrates, such as honey (8005 mOsmol/kg), grape jelly (4555 mOsmol/kg), strawberry jam (4267 mOsmol/kg), orange marmalade (4201 mOsmol/kg) and maple syrup (3758 mOsmol/kg) had the highest osmolalities, which for the most part were close to 10 times higher than those of the liquids and most of the other soft foods. At this point, it should be briefly noted that the approach of measuring a dilution series of the vehicle to extrapolate the osmolality of the undiluted vehicle may possibly lead to a slight overestimation of the osmotic activity of the undiluted preparation, since one creates optimal conditions for the complete dissociation of osmotically active particles by dilution. However, such theoretically possible variations have little effect on the large osmolality differences between soft foods rich in short-chain carbohydrates and other vehicles. It should also be noted that after oral administration, the vehicles mix with the co-administered liquid (usually water) and gastrointestinal secretions and are thereby diluted, which, as shown by the dilution series results, allows for complete dissociation, so the discussion at this point is probably just of theoretical nature.

Surface tension was lowest for peanut butter (31.02 ± 0.60 mN/m), representing the vehicle with the highest fat content, followed by grape jelly (34.09 ± 1.11 mN/m) and grapefruit juice (36.50 ± 0.48 mN/m), which are rich in sugar, and coconut milk (40.09 ± 1.00 mN/m), which again has a high fat content. As for the buffer capacity, there was no direct link between the content and ratio of macronutrients and the surface tension and more detailed information on the composition would be required to possibly derive influences on the surface tension.

While the buffer capacities and osmolalities of some vehicles differed by as much as a factor of ten, the differences in viscosities were even greater. The rheological properties of the soft foods also varied, with most vehicles exhibiting either shear thinning or more Newtonian flow behavior as the shear rate increased. Some vehicles, particularly the purees, exhibited slightly dilatant flow behavior at the onset of the measurements, likely due to food particles that initially had to orient themselves in the shear direction. Dilatant flow behavior was seen particularly for orange marmalade, peanut butter, and strawberries, which can be attributed to a high content of food particles. This is probably also the reason why some vehicles showed rheopex flow behavior with increasing viscosity as a function of time of shear stress. In the case of strawberries, this behavior was so pronounced that it was not possible to measure the viscosity profile at decreasing shear rates. However, the pronounced dilatant flow behavior of the pureed strawberries was most likely due to the gap geometry of the rheometer. It can be assumed that strawberry seeds have wedged themselves here in a way which is very unlikely to be observed *in vivo*. Overall, results obtained when assessing the flow behavior indicate that when a sufficient shear force is applied most of the semisolid vehicles can freely flow. It might be speculative to generally conclude that following dose administration with a semisolid dosing vehicle the shear forces caused by gut motility as well as *in vivo* dilution and digestion of the vehicle will ensure that the drug product is not “entrapped” in the dosing vehicle for a longer time period, but the probability for such an observation seems rather low, since particularly in combination these effects are likely to significantly reduce the vehicle viscosity. The age group for which there is probably the greatest risk for an “entrapment” effect caused by highly viscous vehicles is again the cohort of very young infants, for which, however, one would not as a rule already co-administer medications with semi-solid-, but rather with liquid vehicles. However, if one considers that the drug is mixed with the vehicle prior to administration and that administration does not always take place immediately after mixing as foreseen, then viscosity may well have an influence on the stability and the *in vivo* performance of the dosage form. Low-viscosity liquids can certainly penetrate the dosage form and interact with the drug much more rapidly than the free liquid phase in semi-solid vehicles. However, such effects must be discussed on an individual basis, since several other factors, such as the type of drug product, the composition of the vehicle and possibly other physicochemical properties of the vehicle, are of course also important in this context.

Interestingly, no correlation was observed between vehicle composition and its physicochemical properties. Vehicles belonging to one category of food often differ significantly in their physicochemical properties, as can be seen, for example, in the results for milk and yogurt, both of which belong to the dairy products group. Since the goal of the study was to provide an overview of essential differences in composition and properties of "randomly" selected vehicles of each type, but not to examine variability in composition and properties of individual vehicles, such data are not shown here. However, there are already a few publications that address the properties of further brands of the vehicles listed in Appendix A [5, 6, 13]. Comparing the results of this study with previously published values, variability in physicochemical properties can also be observed within one vehicle type. As discussed earlier, the amount of vehicle used for co-administration and the age of the pediatric patients certainly also determine the extent to which the different properties of the vehicles affect drug stability and *in vivo* performance of different dosage forms. At least for certain dosage form-vehicle combinations, an increasing impact can be expected with very young patient age and with increases in vehicle volume and/or exposure time.

### Current and proposed methodology supporting vehicle selection

The differences in the composition and physicochemical properties of commonly used vehicles reported in this work are important information when estimating the compatibility of pediatric drugs with soft foods and liquids. From all the studies conducted so far, we have learned that in compatibility assessment it is important to look at the composition and physicochemical properties of a vehicle as whole and not to focus on a single parameter, such as pH. In the present study, one commercially available liquid or soft food was investigated as an exemplary vehicle for each of the vehicles listed in Appendix A of the FDA draft guidance to estimate essential differences between the individual vehicles and how physicochemical properties may be linked to their composition. It can however be assumed that composition and physicochemical properties not just vary among different vehicles, but also within one vehicle type, which has already been indicated by the results of some preliminary experimental work. Moreover, even vehicles of the same brand may have different compositions in different countries. Therefore, especially when developing drugs to be approved in many countries around the world, one should not disregard the global availability and the quality of the vehicles listed in the label, since this might pose a fundamental problem regarding a reliable risk assessment. On the other hand, although desirable, it will be impossible to realize the statement in FDA's draft guidance "Assessing the Effects of Food on Drugs in INDs and NDAs—Clinical Pharmacology Considerations" that "all soft foods intended for labeling should be tested" [33]. Compatibility testing with all potential dosing vehicles while considering the variability of each individual vehicle would be a tremendous burden for the sponsor. Moreover, such an effort, which is certainly not feasible, could only be effective if the variability of each vehicle would be known. Nevertheless, a well-designed *in vitro* compatibility test is considered an important tool for assessing product quality, since it will help to reduce the number of *in vivo* studies required for risk assessment and should therefore be implemented by all means. The current FDA draft guidance aims to ensure consistent quality of the drug when administered with a vehicle and indicates the need to standardize the methodology for vehicle selection as well as the preparation and use instructions for the drug-vehicle mixture. However, the focus here is always on one vehicle type, e.g., apple sauce, pudding, etc., without considering how variable its properties can be. Thus, conducting compatibility studies without knowing whether the vehicle chosen for these studies is representative of all vehicles of this type may pose a serious risk. Based on the discussion so far, a better understanding of the variability of individual vehicles should first be sought so that in future compatibility studies a limited number of standardized vehicles which clearly reflect the variability in vehicle composition and physicochemical properties that may be critical to the stability of the drug and the *in vivo* performance of the drug product in question can be used. Critical quality attributes that should be considered are the physicochemical properties highlighted in the present study, i.e. pH, buffer capacity, surface tension, osmolality, viscosity but also the composition of the vehicle, including the carbohydrate:fat:protein ratio, and the calorific content. Depending on the drug under investigation, other vehicle properties such as the presence of metal ions, oxidizing agents and other ingredients that could catalyze drug degradation may also be important. To take all these factors into account in a general test design would certainly go beyond the scope. However, they might be taken into account in product-specific studies if the drug product to be administered contains a drug substance for which such specific stability problems are already known from early development phases. With the aim of establishing a toolbox of standard vehicles that will allow an appropriate risk assessment of co-administering pediatric dosage forms with soft foods and liquids in different locations around the world, the experimental approach of detailed characterization of soft foods and liquids presented here will be pursued and specifically continued with a larger number of representatives for each vehicle type.

## Conclusion

When selecting a vehicle for the administration of oral dosage forms to children, pH is often the most important criterion for ensuring compatibility of drug substance or drug product and vehicle. Results of the investigation of the composition and physicochemical properties of one of each of the vehicles listed in the FDA draft guidance document “Use of Liquids and/or Soft Foods as Vehicles for Drug Administration”, indicate that pH is an important, but not the only parameter that should be considered when evaluating the compatibility of liquids and soft foods used as vehicles for drug administration to children. Vehicles with similar pH can significantly differ in composition and in other physicochemical properties that, when co-administered with oral dosage forms, could impact drug product quality. Moreover, even the pH of individual vehicles can be far more variable than indicated in the draft guidance. Compatibility assessment performed with a certain brand of soft food or liquid will thus not necessarily be predictive for the whole range of marketed products of the same food/fluid type. To identify acceptable (qualified) vehicles for drug product labeling, it is important that the vehicles selected for *in vitro* compatibility screening reflect the variability in composition and essential physicochemical properties of the vehicles recommended on the product label, rather than relying on results obtained with a single vehicle of each type. Furthermore, in order to make the procedure more meaningful with regard to the administration of the drug under investigation to pediatric- as well as other patients around the world and to provide reliable and robust data to both the pharmaceutical developer and regulatory authorities, an appropriate standardization of the *in vitro* method should be considered.
